# The Complex Epidemiological Scenario of West Nile Virusin Italy

**DOI:** 10.3390/ijerph10104669

**Published:** 2013-09-30

**Authors:** Luisa Barzon, Monia Pacenti, Elisa Franchin, Laura Squarzon, Enrico Lavezzo, Margherita Cattai, Riccardo Cusinato, Giorgio Palù

**Affiliations:** 1 Department of Molecular Medicine, University of Padova, Via Gabelli, 63, 35121 Padova, Italy; E-Mails: elisa.franchin@unipd.it (E.F.); laura.squarzon@unipd.it (L.S.); enrico.lavezzo@unipd.it (E.L.); 2 Microbiology and Virology Unit, University Hospital of Padova, Via Giustiniani, 2, 35128 Padova, Italy; E-Mails: monia.pacenti@sanita.padova.it (M.P.); margherita.cattai@sanita.padova.it (M.C.); riccardo.cusinato@sanita.padova.it (R.C.)

**Keywords:** West Nile virus, epidemiology, phylogenetic analysis, surveillance, Italy, mosquitoes, human, horses, neuroinvasive disease, seroprevalence

## Abstract

Entomological, veterinary, and human surveillance systems for West Nile virus (WNV) infection have been implemented in Italy since the first detection of the virus in 1998. These surveillance activities documented a progressive increase of WNV activity and spread in different regions and the emergence of new WNV lineages and strains. Italy is a paradigmatic example of the complex epidemiology of WNV in Europe, where sporadic cases of WNV infection, clusters, and small outbreaks have been reported in several regions. In addition, different strains of both WNV lineage 1 and lineage 2 have been identified, even co-circulating in the same area.

## 1. Introduction

West Nile virus (WNV) is a mosquito-borne flavivirus that belongs to the Japanese encephalitis virus sero-complex. First isolated in Uganda in 1937, the virus was responsible for epidemic outbreaks in Africa and in the Middle East and of sporadic infections in Europe until 1996, when a large outbreak of West Nile neuroinvasive disease (WNND) characterized by a high fatality rate occurred in Romania, followed by other outbreaks and sporadic cases reported in Czech Republic, Southern Russia, and Hungary [1]. The recent years were characterized by the re-emergence of WNV in Europe with human cases of WNND notified in almost all Eastern, Central, and Southern European Countries [2,3]. Italy and Greece have been the most affected EU countries and are reporting human cases of WNND since 2008 [4] and 2010 [5,6], respectively. The virus is also widespread in North America, where it arrived in 1999 [7] and rapidly spread causing hundreds or thousands of human infections each year [8]. While all WNV strains circulating in North America derived from the single introduction that occurred in 1999 [9], European WNV strains probably derive from multiple independent introductions from Africa, followed by local spread and evolution [10,11,12]. 

The complex epidemiology of WNV in Europe is well represented by Italy, where clusters of cases and small outbreaks occurred in different Regions and different strains of both WNV lineage 1 and lineage 2 were identified, even co-circulating in the same area. This review article describes the entomological, veterinary, and human surveillance systems for WNV infection that have been implemented in Italy since the first detection of the virus, and the results of these surveillance activities that documented a progressive increase of WNV activity and spread in different regions and the emergence of new lineages and strains. 

## 2. Methods

A systematic search in the literature was performed for retrieving all available information on WNV surveillance activity and case reporting in Italy. Relevant publications were identified in the PubMed database, and in national and international epidemiological bulletins and reports, *i.e.*, WNV situation updates from ECDC (European Centre for Disease Prevention and Control) [13], epidemiological bulletins from Istituto Zooprofilattico Sperimentale dell’Abruzzo e del Molise G. Caporale, Teramo, Italy [14], updates on WNV from the Italian national epidemiological website EpiCentro [15], bulletins and alerts from the EpiSouth network [16]. The period of publication for literature selection was from 1976 to 2013. In addition, updated information from the Authors is reported.

## 3. Results and Discussion

### 3.1. Surveillance System

A national surveillance system for WNV has been implemented in Italy since 2001, after the first evidence of the presence of the virus provided by the outbreak in horses that occurred in 1998 in the Tuscany Region [17]. The national surveillance system was based on periodic testing of sentinel chickens and horses for seroconversion and entomological surveillance in a defined number of “at-risk areas”, *i.e.*, wetlands that were considered at high risk of WNV introduction because of the presence of a significant number of water fowls including species of migratory birds [2]. The surveillance plan was enhanced with a Ministerial Decree issued on 29 November 2007, which established a rapid alert system, as described [18,19].

Following the notification of the first equine cases of WNND in September 2008, extraordinary surveillance programs for possible human cases of WNV infection were activated in the affected Regions in North-Eastern Italy, as indicated by the national surveillance plan. Surveillance activities included passive surveillance of suspected human cases of aseptic encephalitis and/or meningitis of unknown etiology and active surveillance of WNV infection in subjects at risk because of potential exposure to the vector, e.g., farm workers in stables with WNV-positive animals and household contacts of patients with WNND, as described [20,21,22]. In addition, according to the national surveillance plan, animal and vector surveillance for WNV infection were enhanced in the same areas, with syndromic surveillance of equine cases of WNND, active surveillance of WNV infection in horses, testing for WNV of samples collected from cattle in the affected region as part of sentinel surveillance for bluetongue disease [21], surveillance of WNV infection in sentinel birds and in dead wild birds, capture of mosquitoes in at risk areas and PCR testing for WNV [2,18,23,24]. Human surveillance for WNND started on September in 2008 and on 15 June in 2009 and ended on 31 October. The surveillance period was extended to 15 November and then to 30 November in the following years, according to the observed duration of the periods of high vector activity. 

The regional surveillance programs for WNND in humans were implemented by the Ministry of Health in a National Surveillance Plan in 2010. This program has been updated every year according to epidemiological data and surveillance results [4]. The national plan for human surveillance defines the “area with virus circulation”, where laboratory-confirmed WNV infections in horses or humans had been notified in previous years or during the surveillance period, and the “surveillance area external to the area with virus circulation” that extends for a 20 km radius around the cases occurring in the outermost parts of the area with virus circulation. Extraordinary vector control measures are implemented in areas with virus circulation. Passive surveillance of WNND is carried on in areas with virus circulation and in surveillance areas, while active surveillance of WNV infection in humans is carried on in areas with virus circulation. WNV nucleic acid amplification test (NAAT) screening of blood and hematopoietic stem cells donations is done in affected areas, while solid organ donations are screened in the surveillance areas, according to the blood and transplant directives [25,26]. In addition, at the national level, all blood, tissue and solid organ donors who travelled to an affected area have to be temporarily deferred for 28 days starting with the day they left the affected area [25,26].

In north-eastern Italy, based on the evidence of widespread WNV circulation in the regional territory, Veneto Region also implemented a surveillance plan for West Nile fever (WNF) since 2010. According to this plan, during the surveillance period from 15 June to 30 November, patients with unexplained fever over 38 °C and without leukocytosis are considered possible cases of WNF and are investigated with laboratory tests for WNV infection [27,28]. This surveillance plan is integrated with surveillance of imported and autochthonous cases of dengue and chikungunya and with entomological and veterinary surveillance activities [27].

### 3.2. Surveillance of WNV Infection in Humans

#### 3.2.1. Human Outbreaks of West Nile Disease

Notwithstanding the evidence of the presence of WNV in Italy at least since 1998, with the equine outbreak in horses [17] and subsequent evidence of seroconversions in sentinel animals in different risk areas under surveillance, as detailed in the next sessions, human disease due to WNV infection was not documented for a decade until the first human cases of neuroinvasive disease was diagnosed in 2008. A possible explanation for the absence of human cases could be related to the underestimation of WNV activity and the under-diagnosis of WNV disease in Italy, especially in the years before the first human cases were identified. However, it cannot also be excluded that this was due to the lack of bridge transmission to humans during this decade or to the circulation of less pathogenic strains that did not cause symptomatic disease in humans, before the emergence of a new more virulent strain in 2008. 

The first human cases of WNND and WNF were detected in the Po river area in northeastern Italy in September–October 2008 [20,29], following the alert from the veterinary surveillance that reported equine cases of WNND in the same area [21]. These first human cases included three patients with WNND who were resident in Emilia-Romagna Region [4,29] and one patient with WNND and one with WNF who were resident in Veneto Region [20,30]. Retrospective analysis of CSF samples collected in the Summer 2008 in Veneto Region from patients with aseptic encephalitis or meningitis led to the identification of further four human cases of WNND, with symptom onset in August–September and resident in the same area of WNV circulation [20]. A further five cases of asymptomatic WNV infection, including four residents in the affected area, were identified by active surveillance of farm workers [20]. 

The following year, human cases of WNND were diagnosed in a larger area near the Po river, involving Veneto, Emilia-Romagna, and Lombardy Regions, with a total of 18 confirmed cases of WNND identified in the period from the end of August to the end of September 2009 and two positive organ and blood donors [22,31,32]. These results of human surveillance were in agreement with those from veterinary and entomological surveillance that reported involvement of a territory surrounding the Po river larger than in the previous year, with evidence of WNV spread to western areas [22,23]. 

The year 2010 was characterized by a decrease of WNV activity, in part as a result of effective vector control measures applied in the areas of WNV circulation surrounding the Po river [33]. In fact, in 2010, human cases of infection (three cases of WNND, three of WNF, and two positive blood donors) were detected only in Veneto Region, in areas located north of those affected in 2008 and 2009 [28]. An increasing WNV activity was observed in the following years in these new areas in Veneto Region and in the nearby Friuli-Venezia Giulia Region, with 10 cases of WNND, two of WNF, and six positive blood and organ donors reported in 2011 [4,34] and with occurrence in 2012 of the largest human outbreak ever recorded in Italy, with 25 confirmed cases of WNND, 17 of WNF, and 14 positive blood donors [35,36]. 

In 2013, as of August 31, the epidemiology of WNV in northeastern Italy appears to be changing again. In fact, at least 12 human cases of WNV infection were reported in the Po area that was also affected in 2008–2009, while northern areas were less affected [37]. 

In 2011 and 2012, clusters of WNND were reported also in Sardinia island, with five confirmed and one probable WNND cases recorded in 2011 [38] and two confirmed WNND cases in 2012 [15]. Surveillance in other Italian regions notified a sporadic case of WNF in the Marche Region, Central Italy, in 2011 [39] and a case of WNND in the South of Italy (Basilicata Region) in 2012 [15]. 

These epidemiological data on human cases of WNV infection were in line with the results from entomological and veterinary surveillance that reported WNV circulation and activity in the same areas where human cases were identified. In several cases, entomological and veterinary surveillance could predict the occurrence of human cases by reporting increased vector density and rate of infected mosquitoes and outbreaks in horses [22,27,40,41]. 

In Italy, the onset of WNV disease in humans ranged from late July to late October, with peaks of cases reported in late August and early September. In patients with WNND, the overall percentage of death was approximately 10% and occurred generally in elderly and immunocompromised patients.

#### 3.2.2. Seroprevalence Studies

WNV infection is generally asymptomatic in humans, with neurological symptoms occurring in less than 1% of cases and fever in approximately 10% of cases. Seroepidemiology studies, *i.e.*, detection of specific antibodies against the pathogen in the healthy general population, are useful to determine if a pathogen is circulating in an area or, in areas already affected by outbreaks, to estimate the extent of the epidemics and to better plan intervention strategies. 

A seroprevalence study on WNV infection in humans was performed in Italy in 2006, before the identification of the first human cases of WNV disease. The study, which involved 1,280 subjects belonging to different risk categories resident in Messina, Sicily, did not identify any positivity for antibodies anti-WNV [42]. 

After the detection of the first human cases of disease in September 2008, an intense investigation on WNV activity in Italy was started. Two seroprevalence studies in serum samples collected from blood donors resident in the affected areas in Emilia-Romagna and Veneto Regions in the period from October 2008 to November 2009 reported a mean prevalence of neutralizing antibodies against WNV of 0.7%, with wide variability among different municipalities [43,44]. In 2010, the study was extended to blood donation centres in Veneto Region outside the affected areas, where the prevalence ranged from 0.3% to 1.1% in the different centres [28]. A two-fold increase of IgG WNV seroprevalence was observed in a blood donation centre located in an highly affected area in Rovigo province, Veneto Region, which was included in the study both in 2009 and 2010 [28]. Likewise, a seroprevalence study on blood donors performed in metropolitan Milan in 2009 and in 2011 reported no positive cases in 2009 and a 0.57% seroprevalence in 2011 [45], suggesting an increasing WNV circulation. In line with these data, a relatively high prevalence of antibodies against WNV was demonstrated by a nationwide retrospective survey on 1,248 serum samples collected from solid organ transplant donors in 2009 which showed that 1.2% of the donors were WNV-seropositive [46]. In this study, WNV-seropositivity was recorded in organ donors from several Italian Regions, *i.e.*, Tuscany, Emilia-Romagna, Piedmont-Aosta Valley, Lazio, Friuli-Venezia Giulia, and Basilicata [46], including Regions where WNV activity was documented only by entomological and veterinary surveillance [14].

These seroprevalence data are in line with those reported in other European and Mediterranean countries where WNV outbreaks have been reported, and in the US. The following prevalence data have been reported in serology surveys performed in blood donors: 1.02% in Greece [47], 0.6% in Spain [48], 0.56% in Turkey in blood donors [49], and 1% in the US [50]. The prevalence is higher (5%–15%) in subjects with increased risk of exposure [48,51] and in areas with very high WNV activity [52,53]. 

### 3.3. Surveillance of WNV Infection in Horses, Birds, and Sentinel Animals

#### 3.3.1. Syndromic Surveillance in Horses

**Figure 1:**
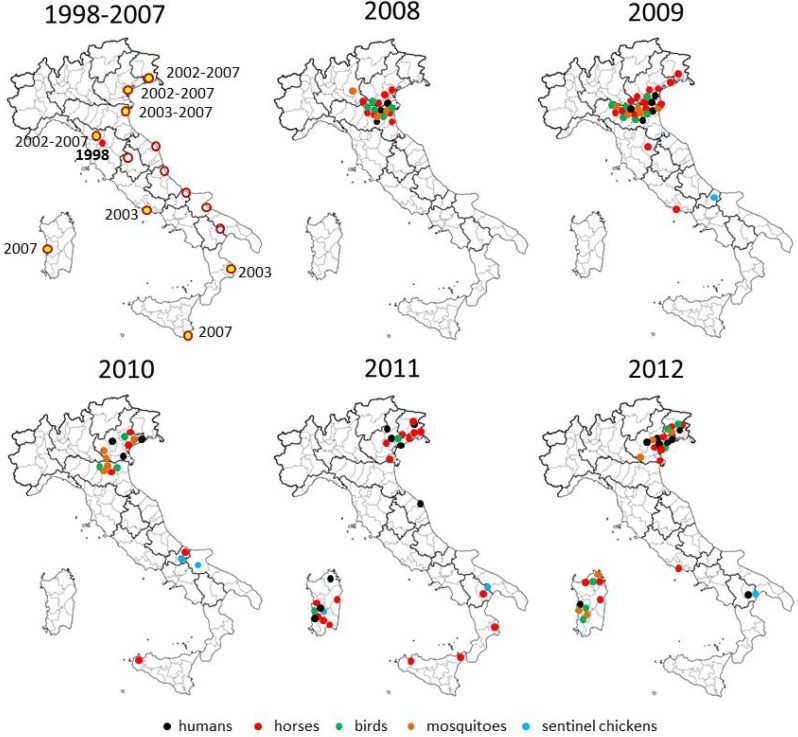
Maps of WNV activity and surveillance results in Italy, 1998–2012. In the map of the years from 1998 to 2007, wetland areas under surveillance for WNV infection are represented with circles; areas where seroconversions of anti-WNV IgGs in sentinel horses and/or chickens were detected are highlighted in yellow and the years reported. In the maps of the years from 2008 to 2012, areas involved by WNV activity, *i.e.*, human and equine cases of WNV disease, WNV detection in birds and mosquitoes, and seroconversion in sentinel chickens, are indicated with dots.

The first outbreak of WNV in horses occurred in the Tuscany Region during the late summer of 1998. During the outbreak, 14 horses tested positive for WNV, with 6 fatal cases, while no infections in humans were recorded [17]. No cases of neuroinvasive disease in horses were reported in the following years, until August 2008, when a new outbreak occurred in the Po river area. In 2008, 273 outbreaks were reported in horse stables in the Po river areas in Emilia Romagna, Veneto and Lombardia Regions, including 563 infected horses out of 1,941 tested (29%). Neurological symptoms were demonstrated in 32 horses, including five fatal cases [18,21]. A wider area was affected in 2009, with a high rate of WNV infection in horses (223 cases of infection in horses, 37 of whom had neurological symptoms and nine died) [19,54] (Figure 1). 

In line with the decreased WNV activity reported by human surveillance, a marked decrease in the incidence of WNV infection in horses was observed in the Po river area in 2010, while outbreaks occurred in Sicily island and in the Molise Region (central Italy), with 88 and 26 cases of WNV infection, respectively [55]. No human cases have been reported so far in Sicily and Molise. Since 2011, equine outbreaks have occurred in Basilicata Region and Sardinia island, where human cases were also reported [56,57]. In particular, a large outbreak with 89 infected horses and 48 horses with neurological symptoms was reported in 2011 in Oristano province, Sardinia [40].

#### 3.3.2. Surveillance in Wild Birds

Serological and molecular testing demonstrated WNV infection in wild birds captured in areas of WNV circulation surrounding the Po river in the period from 2008 to 2010, in line with the results obtained by entomological surveillance. Different species were exposed to WNV infection as documented by the presence of viral RNA in the organs of captured birds and dead birds, including magpies (*Pica pica*), hooded crows (*Corvus corone cornix*), rock pigeons (*Columba livia*), and eurasian jays (*Garrulus glandarius*) [14,19,23,24,33,58]. Since 2010, the virus has been detected in wild birds collected in the areas in northeastern Italy and Sardinia which have been also affected by human cases of WNND [24,33,55,56,57,58,59,60]. Also in these areas, the most affected bird species included magpies, hooded crows, and eurasian jays. At variance with the high mortality observed in the US in some species of the native avifauna, mainly American Crows and, to a lesser extent, American Robins and House Sparrows [61,62,63,64,65], and in Israel in 1998 in Domestic Geese (*Anser anser*) and White Storks (*Ciconia ciconia*) [66], WNV infection was not associated with a significant increase in the mortality of bird species in Italy and in other European countries [19,58,60]. However, the presence of the virus could be demonstrated in dead wild birds collected for surveillance purposes, indicating that the virus could cause disease in some bird species, although they appeared to be less susceptible than the American Crows [19,58,60].

#### 3.3.3. Surveillance in Sentinel Horses and Chickens

After the first WNV outbreak among 14 horses in Tuscany region in 1998 [17], national surveillance activities were implemented in order to detect the circulation of the virus among resident birds, sentinels chickens, and sentinel horses [19]. The surveillance activities carried out from 2001 to 2007 in the 15 Italian wetlands identified by the national veterinary surveillance plan led to the detection of sporadic WNV circulation in several areas through seropositivity and seroconversion in sentinel chickens and horses [2,58] (Figure 1). In the following years, some of these areas became affected by human and equine cases of WNV disease (Figure 1). 

The surveillance in sentinel horses and chickens continued in the following years and included the new areas defined as with virus circulation and of surveillance. WNV detection by PCR, seropositivity, or seroconversion was reported in sentinel animals from areas with virus circulation and sporadically in wetlands under surveillance (Figure 1). 

The surveillance system for WNV in Italy is still focused on the wetland areas identified with the first national surveillance plan in 2002. Some of these areas under surveillance have become affected by human and equine cases of neuroinvasive disease, as shown in Figure 1. These areas are considered at risk because of the high mosquito density and because they are habitat for several species of migratory birds. However, as demonstrated also in other countries with endemic WNV circulation [67,68,69]. WNV activity was observed not necessarily in wetlands. At variance, most cases of infection were reported in rural and residential areas with high density of *Culex* mosquitoes and the presence of susceptible bird species, like passerines and magpies [22,33,57,59]. 

### 3.4. Entomological Surveillance

Although WNV has been isolated from more than 60 species of mosquitoes in North America, only *Culex spp.* and *Aedes/Ochlerotatus spp.* have been significantly implicated in the completion of natural cycle of the virus [70,71]. The first survey on potential vectors for WNV transmission in Italy started in 1999–2002, with the collection of larvae and adult mosquitoes in the Toscana Region, where the equine outbreak occurred in 1998 [17]. Among the 11 species of collected mosquitoes, *Culex spp.* was the most abundant, including *Culex impudicus* and *Culex pipiens* that are competent for WNV transmission [72]. Analysis of mosquitoes pools collected using CO(2)-baited traps in 2008–2009 in the areas in north-eastern Italy where the human and equine outbreak of WNV infection occurred demonstrated the presence of WNV RNA in approximately 1% to 10% of tested mosquito pools, mainly in *Culex pipiens* [22,24,73,74] and rarely in *Ochlerotatus caspius* species [18,19]. In 2010, WNV-positive mosquito pools were detected in the same areas of WNV circulation of the previous years and also in northeastern areas where human and equine cases of WNND were reported [33,55]. At variance, WNV was not detected in other areas in Northwestern Italy not affected by human and equine cases of WNV disease [75]. In 2011, an extensive entomological surveillance in Veneto and Friuli-Venezia Giulia Regions in northeastern Italy detected the presence of WNV in 5 out of 2,732 of *Culex pipiens* mosquito pools examined [41] and, for the first time, demonstrated the presence of both WNV lineage 1 and WNV lineage 2 [59]. In addition, WNV-positive mosquitoes were detected in other Italian Regions, *i.e.*, in Sicily and in Sardinia [56], while no WNV-positivity was documented in the Emilia-Romagna Region, that was highly affected by the WNV outbreak in 2008–2009 [76]. In 2012, both WNV lineage 1 and lineage 2 were detected in mosquitoes collected in northeastern Italy, while WNV lineage 2 appeared to be widespread in Sardinia island [57,60]. The distribution of WNV positivity in mosquitoes is shown in Figure 1. In July and August 2013, WNV lineage 2 was detected in mosquito pools collected in northeastern Italy [77].

### 3.5. Molecular Epidemiology

According to phylogenetic analysis, WNV is classified in lineages and clades. WNV strains that cause disease in humans and horses belong to lineage 1 and lineage 2, while other lineages have been sporadically detected in mosquitoes and birds but not associated with human disease [78]. WNV lineage 1 is composed of strains that circulate in North Africa, Central and Southern Europe, North America, and Australia; WNV lineage 2 contains strains isolated in sub-Saharan Africa and in Madagascar and, since 2004, also in Central and Eastern Europe [79], Russia [80], and Greece (since 2010) [5,6]. 

**Figure 2:**
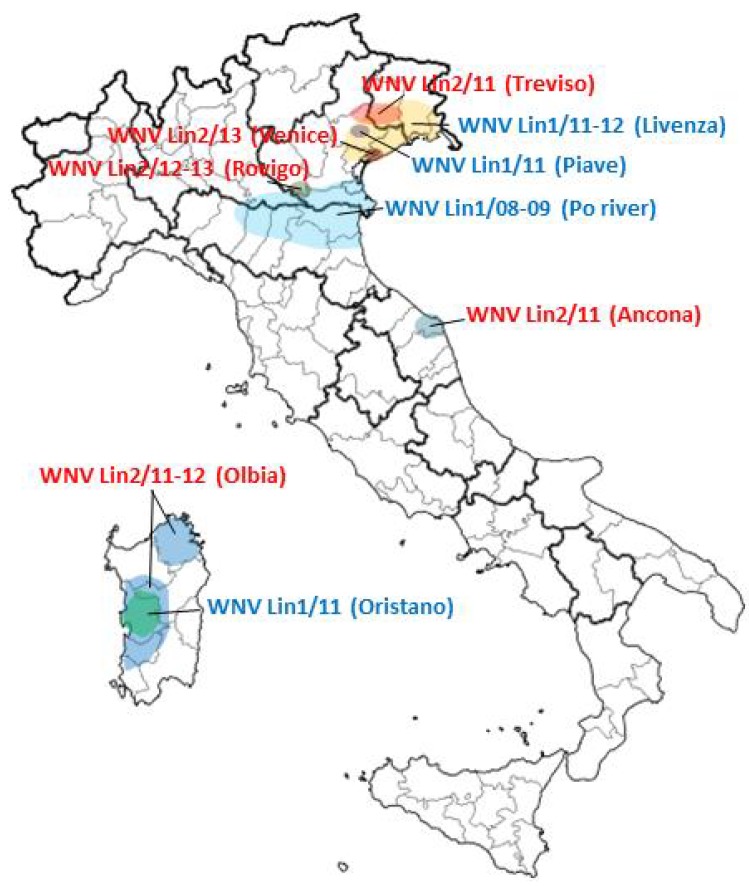
Map of Italy showing the areas where different WNV strains were detected in the period from September 2008 to August 2013. WNV lineage 1 strains are indicated in blue; WNV lineage 2 strains are indicated in red.

**Figure 3:**
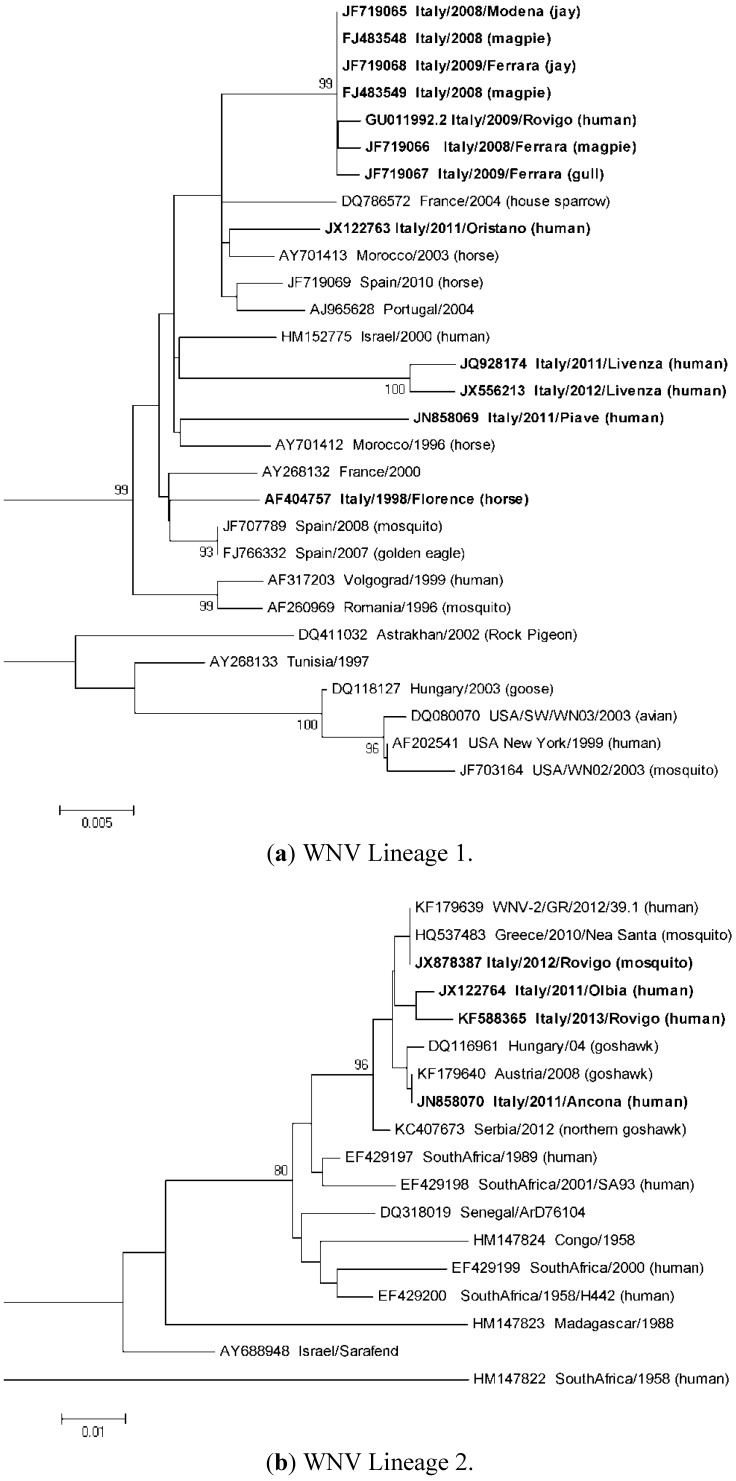
Molecular phylogenetic analysis by Maximum Likelihood method of Italian WNV strains. The evolutionary history was inferred by using the Maximum Likelihood method based on the Tamura-Nei model [83]. The percentage of trees in which the associated taxa clustered together is shown next to the branches. Initial trees for the heuristic search were obtained by applying the Neighbor-Joining method to a matrix of pairwise distances estimated using the Maximum Composite Likelihood (MCL) approach. The trees are drawn to scale, with branch lengths measured in the number of substitutions per site. All positions containing gaps and missing data were eliminated. Evolutionary analyses were conducted in MEGA5 [84]. GenBank accession numbers of the sequences are indicated near the name of WNV strain. Italian WNV sequences are highlighted in bold. (**a**) Maximum Likelihood phylogenetic tree of WNV lineage 1 strains for partial NS5 and 3’UTR genomic sequences (659 nucleotides). The tree with the highest log likelihood (−1635.3077) is shown. The analysis involved 29 nucleotide sequences. (**b**) Maximum Likelihood phylogenetic tree of WNV lineage 2 strains for partial NS5 genomic and 3’UTR sequences (357 nucleotides). The tree with the highest log likelihood (–999.4010) is shown. The analysis involved 18 nucleotide sequences.

Analysis of WNV genome sequences detected so far in Italy demonstrated the succession of different lineage 1 and 2 strains phylogenetically related to the Mediterranean and Eastern European subtypes of lineage 1, clade 1a, or to the Hungarian-Greek lineage 2 strains (Figure 2 and Figure 3). In some cases, old WNV strains were no longer detected after having caused outbreaks and appeared to be displaced by new strains. This could be due to the positive selection of new WNV strains with enhanced fitness and transmissibility, but also to suitable ecological conditions that favored a certain viral strain in a particular niche. 

The lineage 1 isolate that caused the equine outbreak in 1998 in Tuscany was related to WNV strains that were circulating at that time in the Mediterranean basin [17]. This strain was no longer detected and was different from the strain, called Italy/2008–2009, that was responsible of the large outbreaks in humans and horses that occurred in the Po river area in 2008–2009 [12,32,81]. Genome sequence analysis demonstrated high similarity of the viruses isolated in 2008 and in 2009 supporting the hypothesis that the virus had overwintered and become endemic in Italy [19,32,81]. This epidemic strain was detected for the last time in mosquito pools and wild birds collected in the Emilia-Romagna and Veneto Regions in 2010 [33,76,82]. The disappearance of this strain does not seem to be related to vector control measures, since the density of *Culex* mosquitoes even increased in the Po area in 2010, while it could be more probably explained by climatic conditions with drought conditions being a favorable factor that enhance WNV circulation [33]. 

In 2011, a new WNV lineage 1 genome (called Piave from the name of a nearby river in Veneto Region) was responsible for a cluster of human infections from a single solid organ transplant donor who had a recent history of travel in Romania and was also resident in a village where other cases of WNV infection occurred [34,85]. Since this WNV genome sequence has not been identified so far in other cases of infection, neither in Italy nor in other Countries, and phylogenetic analysis was not helpful to define the evolutionary history of the virus, the origin of this virus (imported or autochthonous) remains unknown. 

Another new WNV lineage 1 genome was also sequenced in 2011 in Veneto Region from a blood donor and called Livenza from the name of a nearby river [34]. The Livenza strain was conceivably responsible for the large human outbreak that occurred in the same area in 2012, since it was sequenced and isolated from several human cases of infection and from mosquito pools [35,86]. Also in this cases, the high sequence similarity between the Livenza strains isolated in 2012 with the viral genome fully sequenced in 2011 was consistent with overwintering of the virus [35,86]. 

In 2011, in the same areas in north-eastern Italy, a WNV lineage 2 related to the Hungarian strain was detected in a dead collar dove (*Streptopelia decaocto*) [59], while in 2012 WNV lineage 2 related to the Greek strain was detected in a mosquito pool in a southern site near the Po river [82]. WNV lineage 2 sequences related to the Hungarian-Greek strains were also sporadically detected in 2011 in a patient with WNF from Marche Region (Central Italy) [39] and in a patient with WNND from Olbia (north-eastern Sardinia) [38]. In 2012, this lineage 2 strain was widespread in the Sardinia island, as indicated by its detection in mosquitoes and birds collected in different sites [60]. At variance, the cluster of WNND that occurred in 2011 in a geographically separated area in south-western Sardinia was caused by a WNV lineage 1 strain of the Mediterranean subgroup, related to but different from other lineage 1 Italian strains [38,87]. No WNV lineage 1 was detected in Sardinia region in 2012 [57]. In the summer 2013, at least 7 human cases of WNND and WNF due to WNV lineage 2 related to the Hungarian-Greek strains were identified in the Po river area [37] and circulation of WNV lineage 2 was also demonstrated in mosquitoes (Figure 2) [82].

In most cases, WNV genotypes sequenced in samples obtained by veterinary and entomological surveillance were the same responsible of human cases of disease, with a few exceptions, like the detection of WNV lineage 2 in northern Italy in 2011 and 2012 but no human cases of infection with lineage 2 reported [34,36]. However, patients with WNND or WNF generally have very low viraemia or the virus is no longer detectable in blood at the time of symptom onset. Therefore, in most cases of human infection, information on WNV lineage and genotype is not available. It is conceivable that the number of different WNV strains that have circulated and are currently circulating in Italy and in other European and Mediterranean countries is higher than those that are identified by surveillance systems. Improved molecular methods and the identification of urine as a suitable biological sample where the virus can be detected at high titer for long time [36] have facilitated sequencing of WNV genome from human samples. Therefore, an increasing number of fully sequenced WNV genomes are expected to be available in the future.

### 3.6. Influence of Ecological Conditions

As above reported, sequencing of WNV genome from infected humans, birds and mosquitoes documented persistence of the same viral strains in consecutive years in Italy, supporting the hypothesis of overwintering of WNV in Italy, rather than different introductions [19,23,24,32,33,35,82,86,87]. The mechanism of WNV overwintering has not yet been definitively demonstrated in Italy, since the virus has not been detected in overwintering *Cx. pipiens* mosquitoes [76], while a role of birds has been suggested, based on the identification of infected birds during the spring, when mosquito density was still low [19,74]. Overwintering of WNV has been extensively investigated in the US, where it has been clearly documented to occur in *Culex* mosquitoes [88,89] while the role of birds seems to be less relevant. In fact, prolonged WNV infections, even for months, have been demonstrated to occur in some bird species, but it is still unclear if WNV levels in blood in overwintering birds can sustain transmission through mosquito bite [90,91]. Herd immunity in birds could explain the decline of WNV activity after circulation for two or three years in an area, as occurred in the Po river area after the outbreak in 2008–2009 [76,82] and in Treviso and Venice provinces after the outbreak in 2011–2012 [37]. However, in these areas, the extraordinary vector control measures that were undertaken probably contributed to limit viral circulation. 

### 3.7. Comparison with WNV Epidemiology in Europe

Like in Italy, European countries are also characterized by the circulation of different WNV strains of both lineage 1 and lineage 2 that are causing disease in humans and horses. In addition, other WNV lineages have been identified by veterinary and entomological studies [78,92]. However, genetic information on these strains is still limited and further investigation is needed to fill the gap on the study of the evolution of the viruses that are circulating in Europe. WNV lineage 1 strains have been detected mainly in Western European and Mediterranean countries, such as Portugal, Spain, France, Israel, Tunisia, Marocco, and Italy, where they have been responsible of sporadic infections and small outbreak in human and horses. These viruses are genetically related and grouped in the Western European and Mediterranean cluster [10,11,12]. The Italian WNV lineage 1 strains are included in this cluster, as shown by the phylogenetic analysis (Figure 3(a)). The WNV lineage 1 genotypes that caused the large outbreaks with hundreds of cases of WNND in Romania in 1996 [93] in Russia in 1998–1999 [80,94], and in the US since 1999 [7] are classified in different clusters (Figure 3(a)). In the recent years, pathogenic WNV lineage 2 strains have emerged and are causing human diseases in Europe. The first human outbreaks of neuroinvasive disease due to a WNV lineage 2 were reported in Greece [5] and in Romania and Russia in the Volgograd region in 2010 [95,96]. These outbreaks were caused by two unrelated WNV lineage 2 genotypes that had sequence similarities, respectively, with genotypes detected for the first time in wild birds in Hungary in 2004 [79] and in the Volgograd region in 2007 [97]. WNV lineage 2 strains related to the Greek-Hungarian cluster have recently spread to other European countries, such as Serbia, where a large human outbreak occurred in 2012 [96] and have been detected in Italy [38,39,59,60], as described in the previous section (Figure 3(b)). 

## 4. Conclusions

Like other European and Mediterranean countries, Italy is experiencing the emergence of WNV infections. Since 2008, the virus has been circulating in several regions, mainly in wetland areas, causing small clusters and outbreaks of neuroinvasive disease in horses and humans. The largest human outbreak occurred in 2012 in northeastern Italy, with 25 cases of neuroinvasive disease and 17 cases of fever. Integrated human, veterinary, and entomological surveillance systems have been established and have been successful in the early detection of outbreaks and implementation of vector control measures. Multiple introductions of different WNV lineage 1 and lineage 2 strains have been documented, with evidence of overwintering and establishment of endemic cycles of transmission. 
